# Prompt Placental Histopathological and Immunohistochemical Assessment after SARS-CoV-2 Infection during Pregnancy—Our Perspective of a Small Group

**DOI:** 10.3390/ijms25031836

**Published:** 2024-02-02

**Authors:** Daniela Eugenia Popescu, Ioana Roșca, Ana Maria Cristina Jura, Andreea Cioca, Ovidiu Pop, Nicoleta Lungu, Zoran-Laurențiu Popa, Adrian Rațiu, Mărioara Boia

**Affiliations:** 1 Department of Obstetrics and Gynecology, “Victor Babeş” University of Medicine and Pharmacy, Eftimie Murgu Sq. No. 2, 300041 Timişoara, Romania; 2 Department of Neonatology, Premiere Hospital, Regina Maria Health Network, Calea Aradului, No. 113, 300645 Timişoara, Romania; 3 Faculty of Midwifery and Nursery, University of Medicine and Pharmacy “Carol Davila”, 020021 Bucharest, Romania; 4 Department of Pathology, Premiere Hospital, Regina Maria Health Network, Calea Aradului, No. 113, 300645 Timişoara, Romania; cioca_andre@yahoo.com; 5 Department of Morphological Sciences, University of Oradea, Universității Street, No. 1, 410087 Oradea, Romania; 6 Department XII Obstetrics and Gynecology—Obstetrics and Gynecology III, “Victor Babeş” University of Medicine and Pharmacy, Eftimie Murgu Sq. No. 2, 300041 Timişoara, Romania

**Keywords:** placenta, SARS-CoV-2 infection, pregnancy, vertical transmission, fetal, pathology, immunohistochemistry, vascular complications, COVID-19

## Abstract

Research indicates compelling evidence of SARS-CoV-2 vertical transmission as a result of placental pathology. This study offers an approach to histopathological and immunohistochemical placental observations from SARS-CoV-2-positive mothers compared to negative ones. Out of the 44 examined placentas, 24 were collected from patients with a SARS-CoV-2 infection during pregnancy and 20 were collected from patients without infection. The disease group showed strong SARS-CoV-2 positivity of the membranes, trophoblasts, and fetal villous macrophages. Most infections occurred during the third trimester of pregnancy (66.6%). Pathology revealed areas consistent with avascular villi (AV) and thrombi in the chorionic vessels and umbilical cord in the positive group, suggesting fetal vascular malperfusion (FVM). This study shows SARS-CoV-2 has an impact on coagulation, demonstrated by fetal thrombotic vasculopathy (*p* = 0.01) and fibrin deposition (*p* = 0.01). Other observed features included infarction (17%), perivillous fibrin deposition (29%), intervillous fibrin (25%), delayed placental maturation (8.3%), chorangiosis (13%), chorioamnionitis (8.3%), and meconium (21%). The negative control group revealed only one case of placental infarction (5%), intervillous fibrin (5%), delayed placental maturation (5%), and chorioamnionitis (5%) and two cases of meconium (19%). Our study sheds light on the changes and differences that occurred in placentas from SARS-CoV-2-infected mothers and the control group. Further research is necessary to definitively establish whether SARS-CoV-2 is the primary culprit behind these intricate complications.

## 1. Introduction

Coronavirus disease 2019 (COVID-19) is caused by SARS-CoV-2, also known as severe acute respiratory syndrome coronavirus 2 [1]. When the new SARS-CoV-2 was first discovered in pregnant Chinese women, there was hope that, unlike other RNA respiratory viruses and previous coronavirus infections such as severe acute respiratory syndrome coronavirus (SARS-CoV) and Middle East respiratory syndrome coronavirus (MERS-CoV), vertical transmission would not happen [2]. Recent research indicates that multiple case reports and series have provided compelling evidence of congenital transmission of SARS-CoV-2; as a result, placental pathology is of great interest [3]. 

The maintenance and sustenance of fetal growth depend on the placenta, a special tissue that only forms during pregnancy. It acts as the primary barrier between the mother and her fetus during pregnancy and facilitates the exchange of gases, nutrients, waste products, and hormones [4]. Compared to the cytotrophoblast and other cells within the chorionic villus core, which are extremely prone to infections, the syncytiotrophoblast layer, which comes into direct contact with maternal blood, is fairly resistant to most pathogens [5]. The extravillous trophoblast cell layer of the membranous chorion comprises the outer wall which surrounds approximately 60–70% of the surface. Apart from the membranes’ structural barrier, trophoblasts use the Toll-like receptor TLR4 to identify bacterial products and signals nearby immune cells to preserve tolerance. Their role is to support innate immunity alongside the amnion by generating antimicrobial peptides to ward off infection [6]. 

Cell entry and the spread of SARS-CoV-2 are widely thought to depend on the angiotensin-converting enzyme 2 (ACE2) receptor and the serine protease TMPRSS2 [7]. In both the first and second trimesters of pregnancy, placentas exhibit a notable expression of each entry receptor. Lower levels of entry cofactor expression are seen in the placenta and chorioamniotic membranes at the maternal–fetal interface at term [8].

Often referred to as “SARS-CoV-2 placentitis”, the most common histopathological triad is represented by chronic histiocytic intervillositis (CHI), villous trophoblast necrosis, and intervillous fibrin deposition (IFD) [9]. Other studies show that more frequent features in the placentas of infected women are represented by maternal and deciduous thrombosis, increased intervillous fibrin, and, in rare cases, fetal thrombosis [10].

The paramount objective of this study is to meticulously compare both histopathological and immunohistochemical observations of placentas from mothers who have tested positive for SARS-CoV-2 during their pregnancy with those who tested negative for infection throughout the gestational period. By undertaking this comprehensive analysis, we aim to shed light on any potential disparities or unique characteristics that may emerge from such a comparison, thus contributing significantly to our understanding of the impact of this relentless virus on maternal health and fetal development. 

## 2. Results

The positive group consisted of 24 patients aged 21–38 years (mean 31.37 years). The SARS-CoV-2 patients gave birth between 37 and 41 weeks gestational age (mean 38.91 weeks). Their newborns had birth weights ranging from 2950 to 3800 g (mean 3333.75 g) and no associated comorbidities. 

Maternal symptoms and treatment from the positive group were divided into several categories, as described in Table 1. All 24 mothers received vitamin therapy (vitamin D and C), ensuring appropriate supplementation during the infection period. An impressive percentage of these mothers also received antipyretics, with 19 out of 24 (79.1%) benefitting from this effective method of reducing fever and alleviating discomfort.

Interestingly, while anticoagulant medication was not as widely administered, it played a crucial role in the treatment of a select few patients. Only four patients (16.6%) required such medication to manage their condition effectively. When it came to identifying the most prevalent signs of the disease, flu-like symptoms were dominant among the affected mothers. A staggering 14 out of 24 (58.3%) experienced symptoms such as fatigue, chills, pharyngitis, headaches, rhinorrhea, and myalgia.

Furthermore, it is worth noting that a considerable number of patients developed a fever during their battle against this infection. Approximately 29.1% (seven patients) experienced this symptom as part of their illness progression. Intriguingly enough, only a small fraction of patients presented with anosmia which affected merely three individuals (12.5%). This distinctive COVID-19 symptom, together with the ageusia observed in five patients (20.8%), highlights the diverse range in how this infection manifested itself among different individuals.

The majority of SARS-CoV-2 maternal infections occurred during the third trimester of pregnancy, accounting for 66.6% (16 out of 24 cases). Additionally, 25% (six cases) occurred during the second trimester and 8.3% (two cases) occurred during the first trimester. This period of infection emphasizes the importance of continuous vigilance and appropriate precautions throughout all stages of pregnancy in order to safeguard maternal and fetal health against this highly infectious virus.

The main characteristics of the control group are described in Table 2. The negative group consisted of 20 patients aged 20–40 years (mean 32.27), with a mean gestational age of 39.2 weeks and a mean birth weight of 3250.56 g, with no associated comorbidities or complications during pregnancy or birth. All pregnancies were followed-up by specialized obstetricians capable of detecting any minor change or alterations in the fetal status. No infections were detected during the gestational period, and no associated pathologies that could alter the placental circulation occurred, such as pregnancy-induced hypertension, gestational diabetes, and cardiac or pulmonary disfunctions.

### 2.1. Macroscopic Observations

**Figure 1:**
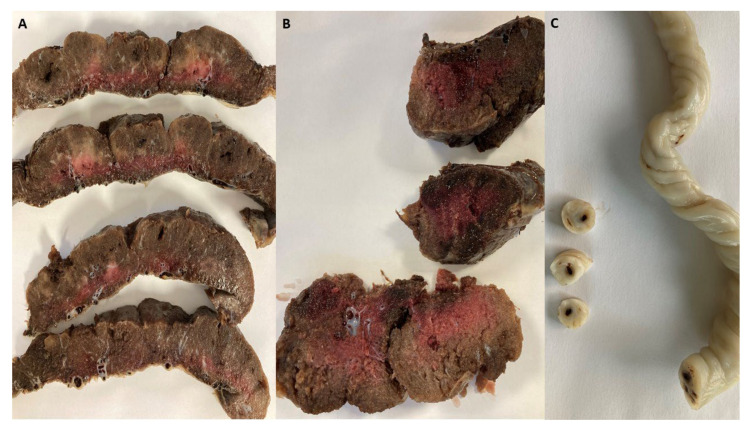
(**A**) Macroscopic image of placentas, serially sectioned, showing pale areas consistent with avascular villi; (**B**) thrombi in the chorionic plate vessels and stem villous vessels; (**C**) umbilical vein containing a thrombus.

We conducted a meticulous macroscopic examination of the placentas of both groups. Initially, the placentas from the disease group were assessed and striking observations were made that shed light on the underlying factors contributing to the fetal vascular malperfusion (FVM) phenomenon. A macroscopic evaluation revealed distinct regions characterized by avascular villi (AV), a clear indication of a compromised blood flow within these vital structures. Additionally, thrombi were observed in both the chorionic vessels and the umbilical cord, as seen in Figure 1, further affirming the presence of FVM.

In the positive group, a remarkable finding was observed where fetal thrombotic vasculopathy was detected in an astounding 29% of cases, in stark contrast to the placentas from the control group. The statistical significance of this discrepancy was confirmed with a *p*-value of 0.01. This compelling evidence strongly suggests that SARS-CoV-2 infection during pregnancy exerts a significant impact on coagulation disorders. 

**Figure 2:**
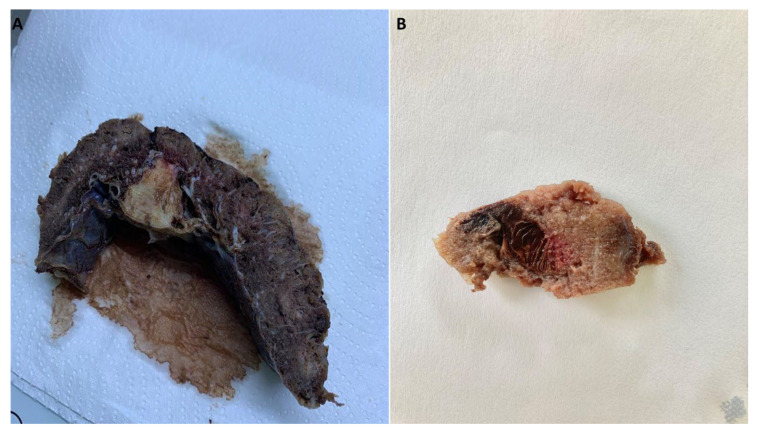
(**A**) Macroscopic image of an old intervillous thrombus and (**B**) a recent intervillous thrombus showing angular contours and lines of Zahn.

Intervillous thrombosis, as illustrated in Figure 2, was noted to be present in a significant proportion, accounting for approximately 21% of the cases. This finding is particularly noteworthy when compared to the control group. However, the difference was not statistically significant (*p* = 0.053). Thrombi exhibit a distinctive feature known as lines of Zahn, which can be observed in Figure 2. These lines consist of lighter layers of platelets and fibrin, as well as darker layers of red blood cells.

### 2.2. Microscopic Observations

Through our comprehensive study, we have uncovered an abundance of microscopic involvement that exhibits a strong correlation with the visible placental appearances. The examination of these microscopic details has provided us with invaluable insights, enabling us to better understand the underlying mechanisms at play within this vital organ. Microscopic observations were correlated between groups and can be seen in Table 3.

**Figure 3:**
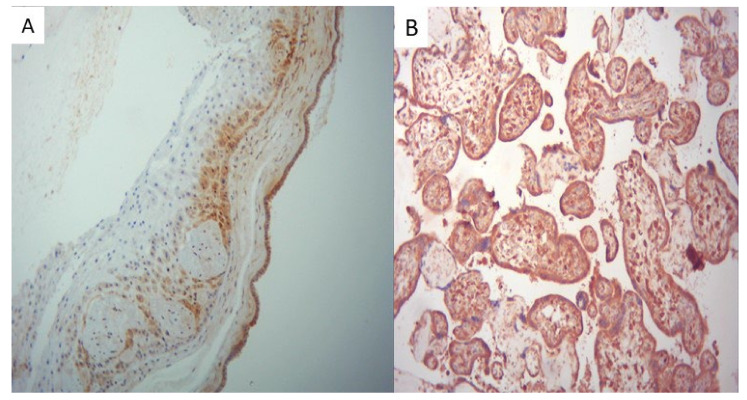
(**A**) Immunohistochemistry for the SARS-CoV-2 protein showing strong positivity of the placental membranes (×10) and (**B**) of the trophoblast and fetal villous macrophages (×20).

All placentas from the positive group exhibited strong positivity for SARS-CoV-2 in the placental membranes, trophoblast, and fetal villous macrophages, demonstrated in Figure 3. 

**Figure 4:**
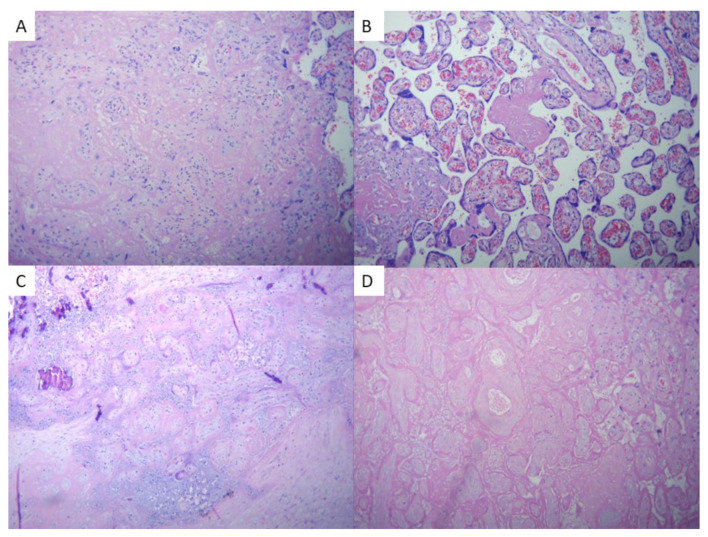
(**A**) Massive perivillous fibrin deposition; (**B**) A representative field of a placenta with chorangiosis; (**C**) recent infarct showing collapsed villi with loss of nuclear basophilia; (**D**) old infarct showing pale ghosts of necrotic villi (×20, hematoxylin and eosin).

**Figure 5:**
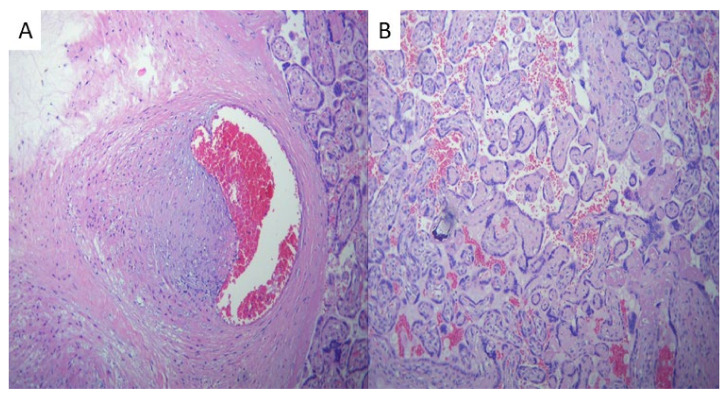
(**A**) Microscopic image of a nonocclusive thrombus showing a fibrin thrombus cap; (**B**) A field of avascular in the center of the image showing villi with eosinophilic and paucicellular stroma without capillaries (×20, hematoxylin and eosin).

The COVID-19 placentas exhibited a multitude of significant features that shed light on the impact of the virus on placental health. These features, such as placental infarction, perivillous fibrin deposition, intervillous fibrin, delayed placental maturation, chorangiosis, chorioamnionitis, and meconium presence, provide crucial insights into the intricate complexities of this condition and can be observed in Figure 4 and Figure 5.

Among these features, placental infarction was observed in 17% of cases in the COVID-19 group compared to only 5% in the negative control group (*p* = 0.4). This notable difference underscores the potential influence of COVID-19 on vascular complications within the placenta. Furthermore, perivillous fibrin deposition was found in approximately 29% of COVID-19 placentas. This observation suggests that COVID-19 may trigger an abnormal thrombotic response within the maternal–fetal interface. In stark contrast, no such findings were documented in the comparative group, highlighting a statistically significant difference (*p* = 0.01).

Intervillous fibrin was also present in about 25% of COVID-19 placentas. This finding indicates potential disruptions to blood flow and nutrient exchange between mother and fetus due to SARS-CoV-2 infection. Delayed placental maturation was observed in a small percentage (8.3%) of COVID-19 placentas. This delay may have implications for fetal development and overall pregnancy outcomes; however, the small percentage of this type of lesion is a good indicator of good neonatal outcomes. 

Chorangiosis, characterized by an increased number and dilation of fetal vessels within chorionic villi, was seen in approximately 13% of cases affected by COVID-19. This finding points towards possible alterations in normal vascular development within the placenta due to viral infection. Additionally, chorioamnionitis—inflammation of both the chorion and amnion—occurred at a rate of 8.3% among placentas affected by COVID-19 and not at all in the control group (8.3% vs. 0%, *p* = 0.2). The presence of this inflammatory response suggests possible maternal immune system activation triggered by viral infection.

Lastly, meconium staining was observed in five cases in the positive group (21%) and two in the negative group (10%) (*p* = 0.4). The presence of meconium, which is a sign of fetal distress, may indicate potential adverse effects on the fetus due to COVID-19 infection.

Together, these findings provide compelling evidence regarding the impact of COVID-19 on placental health and pregnancy outcomes. They highlight the need for further research and understanding of the virus’s influence on maternal–fetal health to ensure optimal care and management for affected individuals.

## 3. Discussion

The intriguing correlation between placental pathology and maternal SARS-CoV-2 infection has sparked great interest among obstetricians, histopathologists, and dedicated scientists who are committed to studying the profound impact of SARS-CoV-2 infection on pregnancy outcomes [11]. Understanding the intricate relationship between these factors holds immense potential for enhancing our knowledge and guiding clinical management in this ever-evolving field of research.

The objective of our study was to meticulously compare the histopathological and immunohistochemical findings in placentas from pregnant mothers who tested positive for SARS-CoV-2 during pregnancy with those who tested negative for infection throughout the entire pregnancy.

The infection primarily manifested during the third trimester of pregnancy (66.6%), with the majority of cases presenting flu-like symptoms followed by fever. Treatment involved administering vitamin therapy and antipyretics, and a small percentage of patients received anticoagulants. Sessa et al. describes in a study that out of the 68 women who tested positive for SARS-CoV-2, 35.29% were symptomatic while 64.71% were asymptomatic [12]. 

In the positive group, fetal thrombotic vasculopathy was seen in 29% of cases compared to the placentas from the control group, suggesting SARS-CoV-2 infection during pregnancy has a great impact on coagulation disorders, although coagulation can already be altered by pregnancy itself [13]. One study found thrombo-hemorrhagic alterations, with decidual microvascular thrombi being present in three cases, a significantly different frequency compared to the control cohort (*p* = 0.003) [14]. Glynn et al. describes that FVM lesions appeared more frequently among acute SARS-CoV-2 than either nonacute SARS-CoV-2 or control cases (53.8% vs. 18.8% vs. 13.2% for acute, nonacute, and controls, respectively; *p* < 0.001) [15]. Furthermore, FVM was observed in 27.08% of cases (95% CI, 19.2−35.6) in another study conducted by Di Girolamo et al. [16].

Intervillous thrombosis (Figure 2) was also noted during the evaluation of the placentas of SARS-CoV-2-infected patients, consisting of 21% of cases compared to the control group. Bouachba et al. described large intervillous thrombi that were disseminated throughout all the placental parenchyma and were not only confined to the basal plate [17].

Our study revealed extensive microscopic involvement correlating with the gross placental appearances, similarly to the ones described by Fitzgerald et al. [18].

Histopathological examination of the placentas revealed a spectrum of pathologically increased perivillous fibrinoid deposition counting for seven of the positive group (29%), versus none in the comparative group (*p* = 0.01). Zaigham et al. found massive perivillous fibrinoid deposition (MPFD) in 13 of the 14 placentas (93%) [3]. Argueta et al. observed that the intervillous spaces showed extensive infiltration of maternal immune cells, perivillous fibrin deposition, and clots with erythrocytes, mononuclear cells, and fibrin deposition, while none of the negative samples or negative controls displayed massive chronic intervillositis (MCI), chronic histiocytic intervillositis (CHI), or infiltration of maternal immune cells [8].

Chronic placental hypoperfusion or low-grade tissue hypoxia was also a concern among pregnant patients with COVID-19 disease during pregnancy. Our study revealed three cases of chorangiosis (13%) in the positive group with none in the negative group, with similar data in the literature. Ferraiolo et al. also observed that the terminal villi of the placentas collected from SARS-CoV-2-infected mothers were characterized by capillary congestion and focal microchorangiosis [19]. Chorangiosis was also described by Levitan et al., but was not among the most prevalent characteristics for this group of patients [1]. Hsu et al. describes an overall histology consistent with acute uterine hypoxia (subchorionic laminar necrosis) superimposed on chronic uterine hypoxia (extra-villous trophoblasts and focal chronic villitis), of which hypertrophic arteriolopathy may be partly responsible [20].

Placental infarction, resulting from the interruption of blood supply, was seen in four of the positive group placentas (17%) and in only one case of the control group (5%). In an article written by Salvatore et al., placental infarcts in different stages of organization were observed in 192 samples (19.8%) [9]. This observation is different from that of Levitan et al., who found that villous infarction was more common in the SARS-CoV-2-negative group (16% of the cases), indicating that this feature is occasionally seen in pregnancies with COVID-19 disease [1]. However, the authors included patients with diabetes mellitus, preeclampsia, and pregnancy-induced hypertension in the control group, which was not the case in our study.

Intervillous fibrin occurred in 6 of the 24 studied placentas in the positive group (25%), compared to one case in the negative control group (5%). Reagan-Steiner et al. noted increased villous fibrinoid necrosis as a feature of villous trophoblastic injury in the placentas of SARS-CoV-2-infected mothers [21].

Meconium staining of the placenta was seen in five cases in the positive group (21%) and two in the negative group (10%). Glynn et al. describe different distributions of the frequency of meconium staining of the placenta across three studied groups (65.4% vs. 40.6% vs. 36.2% for acute, nonacute, and controls, respectively; *p* = 0.018) [15].

Examination of placentas collected from SARS-CoV-2-positive mothers during pregnancy revealed that, while the features on the slides varied, they shared a common pattern, in accordance with findings described in the literature, such as perivillous fibrin deposition, aggregation of villi with obliteration of the intervillous space, and fetal thrombotic vasculopathy.

Despite the mild to moderate symptoms and favorable outcome of our study group of positive patients, there is no question about the importance of SARS-CoV-2 vaccination and the subsequent immunity it provides [22,23].

One of the key challenges we faced in our study was the limited sample size, largely due to the time constraints we encountered when collecting, preparing, and analyzing placental data. This constraint ultimately impacted the overall scope of our research. However, despite this limitation, we were able to gather valuable insights and draw meaningful conclusions from the data that were available to us. Due to the limited testing methods used, which only included PCR and rapid SARS-CoV-2 testing, the assessment of COVID-19 infection in the control group did not include the evaluation of SARS-CoV-2 antibodies. This omission may introduce potential bias into the results.

## 4. Materials and Methods

We examined 44 placentas from patients who delivered between November 2021 and August 2022, collected from mothers who were not vaccinated against SARS-CoV-2 infection before/during pregnancy. All patients were investigated and gave birth in our clinic, the Premiere Hospital of the Regina Maria Private Health Network, Timișoara, Romania. We divided them into two groups: The first was the positive one, consisting of 24 placentas which were collected and inspected from SARS-CoV-2 positive patients (confirmed using the polymerase chain reaction (PCR) technique). In these cases, the infection was acquired during pregnancy or at birth. Inclusion criteria were represented by COVID-19 confirmation using the PCR technique. Individuals vaccinated prior to or during pregnancy with a SARS-CoV-2 vaccine were excluded from the study, along with those suffering from pathologies that could impact placental blood circulation, such as pregnancy-induced hypertension, diabetes mellitus, and signs of impaired fetal growth. Furthermore, we excluded preterm births from the study. Mothers who tested negative for COVID-19 during their pregnancy and did not have any other associated infectious pathologies made up the control group. For this group, the inclusion criteria were defined based on negative SARS-CoV-2 symptoms and tests during pregnancy. The same exclusion criteria were applied for the second group as a way to rule out bias; vaccination against SARS-CoV-2 before or during pregnancy and other pathologies that could interfere with placental blood flow were an argument for exclusion. 

Clinicopathological data and macroscopy pictures were obtained from the database of the Premiere Hospital of the Regina Maria Private Health Network, according to the protocols approved by the ethics committee (No. 330/18.11.2021) that met the standards of the World Medical Association Declaration of Helsinki. 

Tissue samples were fixed in 10% buffered formalin for a minimum of 48 h and then embedded in paraffin. Placentas were weighed after formalin fixation and after removal of amniotic membranes and the umbilical cord. The sections required for histological evaluation were obtained from the umbilical cord and amniotic membranes, and some included the entire thickness of the placental parenchyma. Subsequently, segments were made from 5 μm-thick paraffin blocks and slides were stained using standard hematoxylin–eosin stain. 

To perform the immunohistochemistry technique, representative components were taken from 3 μm-thick placentas. During the first phase, standard deparaffinization and dehydration of the sections were performed, followed by heat-induced epitope retrieval for 20 min with citrate buffer at a pH of 6.0. Endogenous peroxidase blocking was performed with 3% hydrogen peroxide for 5 min. Incubation with primary monoclonal antibody specific for SARS-CoV-2 nucleocapsid, at a dilution of 1:50 (model STJ11101210, St John’s, UK), lasted 30 min. A NovoLink Max Polymer Detection System was used for visualization. Counterstaining was performed using modified Lillie’s Hematoxylin for 5 min. The entire immunohistochemical procedure was performed using a Leica Bond-Max Autostainer (Leica Biosystems, Buffalo Grove, IL, USA). 

Microscopic evaluation was performed using a Nikon Eclipse E600 microscope (Nikon, Tokyo, Japan) and images were acquired and analyzed using Lucia G software 4.7.

Placentas collected from patients confirmed to have SARS-CoV-2 using the PCR technique during pregnancy were considered positive controls and placentas collected from patients who did not contract the infection during pregnancy were considered negative controls. 

### Statistical Tests

Statistical analysis was performed using Fisher’s exact test to determine the relationship between SARS-CoV-2 and clinical parameters and histological changes. Values less than 0.05 were considered statistically significant. Statistical tests were performed in RStudio for Mac, version 2023.09.1 + 494. Categorical data are summarized as counts and percentages. *p*-values were computed to evaluate the degree of correlation between the variables for each individual predictor. A statistically significant *p*-value was defined as one that was less than 0.05.

## 5. Conclusions

This paper explored the changes that occurred in placentas from SARS-CoV-2-infected mothers during pregnancy compared to a control group. Our study sheds light on the stark differences they exhibit. Through meticulous research, we have uncovered the impact of SARS-CoV-2 on coagulation, which manifests through fetal thrombotic vasculopathy and the deposition of fibrin. It is important to note that while pregnancy itself can influence coagulation, our findings strongly suggest that SARS-CoV-2 plays a significant role in inducing vascular and circulatory pathologies during this delicate stage of life. However, it is vital to emphasize that further research is necessary to definitively establish whether SARS-CoV-2 is the primary culprit behind these intricate complications. Only through such investigations can we unlock invaluable insights that could pave the way for enhanced medical interventions and ultimately safeguard the well-being of pregnant individuals.

## Figures and Tables

**Table 1:** Characteristics of mothers from the SARS-CoV-2 infection group (*n* = 24).

Characteristics	Variables	*n*	%
Gestational age	37	1	4.1%
	38	7	29.1%
	39	11	45.8%
	40	3	12.5%
	41	2	8.3%
Gravidity and parity *	G I P I	14	58.3%
	G II P II	9	37.5%
	G IV P III	1	4.1%
COVID-19 disease during pregnancy	First trimester	2	8.3%
	Second trimester	6	25%
	Third trimester	16	66.6%
Type of birth	Cesarean section	15	62.5%
	Vaginal birth	9	37.5%
Maternal symptoms	Flu-like symptoms **	14	58.3%
	Anosmia	3	12.5%
	Ageusia	5	20.8%
	Fever	7	29.1%
Treatment	Vitamin therapy	24	100%
	Anticoagulant	4	16.6%
	Antipyretics	19	79.1%
	Antibiotics	2	8.3%
Associated pathology	Hypothyroidism	1	4.1%
	Thrombophilia	1	4.1%

* G = gravida, P = para, ** fatigue, chills, pharyngitis, headaches, rhinorrhea, myalgia.

**Table 2:** Characteristics of mothers from the control group (*n* = 20).

Characteristics	Variables	*n*	%
Gestational age	37	2	10%
	38	6	30%
	39	5	25%
	40	5	25%
	41	2	10%
Gravidity and parity *	G I P I	12	60%
	G II P II	6	30%
	G III P III	2	10%
Type of birth	Cesarean section	9	45%
	Vaginal birth	11	55%
Associated pathology	Hypothyroidism	2	10%
	Thrombophilia	1	5%
	Anemia	3	15%
	Depression	1	5%

* G = gravida, P = para.

**Table 3:** Histopathological examination of the placentas, group comparison.

Characteristic	COVID-19 Placentas, *n =* 24 ^1^	Control Group, *n =* 20 ^1^	*p*-Value ^2^
Placental infarction	4 (17%)	1 (5%)	*0.4*
Perivillous fibrin deposition	7 (29%)	0 (0%)	*0.011*
Intervillous fibrin	6 (25%)	1 (5%)	*0.11*
Intervillous thrombosis	5 (21%)	0 (0%)	*0.053*
Delayed placental maturation	2 (8.3%)	1 (5%)	*>0.9*
Placental chorangiosis	3 (13%)	0 (0%)	*0.2*
Chorioamnionitis	2 (8.3%)	1 (5%)	*>0.9*
Meconium	5 (21%)	2 (10%)	*0.4*
Fetal thrombotic vasculopathy	7 (29%)	0 (0%)	0.011

^1^ *n* (%); ^2^ Fisher’s exact test.

## Data Availability

Data is contained within the article.
